# Integrating PROMIS measures into an interventional spine workflow: a quality improvement study

**DOI:** 10.1186/s41687-026-01152-8

**Published:** 2026-07-20

**Authors:** Cole Cheney, Martha Springsted, Tejaswini Pisati, Jason Dauffenbach

**Affiliations:** 1 Department of Pain Medicine, Mayo Clinic Health System, Mankato, USA; 2 Spine Center, Mayo Clinic, Rochester, USA

**Keywords:** Spine, Pain, Interventional, Outcomes

## Abstract

**Objective:**

To evaluate and improve the integration of Patient-Reported Outcomes Measurement Information System (PROMIS) data collection within an interventional spine workflow, and to assess feasibility through completion rates before and after a structured workflow intervention.

**Design:**

Retrospective quality improvement study with pre-post intervention analysis.

**Setting:**

Academic community-based health system.

**Subjects:**

Seventy-one unique patients (73 procedures) treated with BVNA between 2022 and 2025.

**Methods:**

PROMIS Pain Interference, Pain Intensity, and Physical Function surveys were administered at baseline and at 3-, 6-, and 12-month follow-up using EHR-embedded automated workflows. In parallel, a structured quality improvement intervention was implemented to improve survey completion via emphasis on baseline survey completion. The intervention included a standardized nursing rooming checkpoint to verify PROMIS completion prior to the procedure, staff education with scripted patient engagement language, real-time EHR visual prompts using dot phrases to identify incomplete surveys, and monthly site-level performance dashboards reviewed with clinical staff and leadership. Completion rates were extracted from institutional dashboards and workflow bottlenecks were identified through process mapping and staff feedback. Fisher’s exact test was used to compare pre- and post-intervention baseline PROMIS completion rates. Clinic visit duration was tracked as a balancing measure to evaluate potential workflow burden.

**Results:**

Seventy-one patients underwent 73 BVNA procedures. Completion rates were 31.82% at baseline, 77.78% at 3 months, 40.00% at 6 months, and 100% at 12 months among patients reaching those timepoints. Longitudinal engagement was 56%, while longitudinal completeness was 33%. Baseline survey completion improved from 31.82% (21/66) before intervention to 100% (6/6) following implementation during the post-intervention three month observation period. Clinic workflow efficiency was not adversely affected, with 83% of visits remaining within the standard 30-minute time slot and no statistically significant increase in visit duration.

**Conclusions:**

PROMIS data collection during interventional spine care is feasible and can be reliably integrated into routine clinical workflows when supported by targeted quality improvement interventions. Improving baseline survey capture potentially expands the denominator of patients eligible for longitudinal outcome measurement without increasing clinical workload. Hybrid implementation strategies combining automated EHR deployment with structured clinical oversight may enable reliable PROMIS integration in procedural spine practice.

## Introduction

Routine collection of patient-reported outcome measures (PROMs) is an essential component of outcomes research and quality improvement. The Patient-Reported Outcomes Measurement Information System (PROMIS) provides validated, domain-specific tools that can be integrated into electronic health records (EHRs) to track pain, function, and well-being over time.

Despite widespread institutional implementation, integrating PROMIS into procedural workflows remains challenging. In collaboration with our institutional outcomes analyst, PROMIS surveys were successfully triggered for patients undergoing outpatient interventions such as basivertebral nerve ablation (BVNA). However, patient nonresponse remained a major barrier, resulting in incomplete data capture and limiting the usefulness of PROMIS for evaluating real-world procedural outcomes.

Clinical trials have established the efficacy of BVNA using legacy measures such as the Oswestry Disability Index (ODI) and Visual Analog Scale (VAS). However, PROMIS outcomes have not been systematically reported in this population. Smuck et al. (2021) included PROMIS Global Health metrics in a large real-world cohort, but because that study was conducted as a clinical trial, patients were contacted directly for follow-up, facilitating high completion rates. In contrast, routine procedural workflows within health systems rely on automated survey deployment, where provider awareness, patient engagement and portal participation, as well as EHR limitations largely determine response rates.

This study was conducted as a quality improvement (QI) initiative designed to optimize PROMIS data capture within a BVNA workflow. We aimed to [1] determine completion rates at defined intervals [2], identify workflow barriers to survey completion, and [3] provide recommendations for improving PROMIS integration in interventional spine care and [4] implement said recommendations. We hypothesized that introducing structured workflow checkpoints, real-time EHR prompts, and performance feedback would improve PROMIS completion by addressing known barriers in provider awareness, patient engagement, and survey visibility.

In parallel with feasibility assessment, this work was conducted as part of a formal quality improvement initiative. Early feasibility findings were used to design and implement workflow interventions intended to improve PROMIS baseline capture and sustain longitudinal follow-up.

## Methods

### Study design and population

This retrospective quality improvement study included all patients who underwent basivertebral nerve ablation (BVNA) performed by five physicians within a single academic community health system from 2022 to 2025.

Cases were identified using an electronic health record (EHR) query tool. Seventy-one unique patients underwent 73 BVNA procedures; two patients were treated twice on different dates. Although 73 procedures were performed, only 66 resulted in a new Tier 4 series assignment and were therefore included in the denominator for completion-rate calculations. The remaining 7 procedures occurred in patients who were already enrolled in an active Tier 4 series at the time of the BVNA procedure. Under the current Tier 4 workflow, once a patient is assigned to a series, the series remains active for its full duration unless manually discontinued. As a result, subsequent BVNA procedures performed during an active series do not trigger a new series assignment and are not separately tracked by the current automation. These 7 procedures were therefore excluded from the series-assignment denominator.

### Outcomes and data collection

Within this BVNA population, 8 PROMIS profile domains (PROMIS^®^ Item Bank v2.0 – Ability to Participate in Social Roles and Activities, PROMIS^®^ Item Bank v1.0 – Emotional Distress – Anxiety, PROMIS^®^ Item Bank v1.0 – Emotional Distress – Depression, PROMIS^®^ Item Bank v1.0 – Fatigue, PROMIS^®^ Item Bank v1.1 – Pain Interference, PROMIS^®^ Item Bank v2.0 – Physical Function, PROMIS^®^ Item Bank v. 1.0 – Sleep Disturbance, PROMIS^®^ Numeric Rating Scale v.1.0 – Pain Intensity 1a) as well as an opioid-use questionnaire are assigned electronically at all longitudinal intervals. The institutional dashboard’s completion rate definition focused on 3 of these measures; meaning, if the patient completed at least PROMIS Pain Interference, Pain Intensity, and Physical Function, the interval was considered complete for the purpose of calculating completion rates.

PROMIS deployment followed an institutional surgical series that anchored to procedural events such as BVNA. Baseline surveys were automatically assigned at the time of procedure scheduling and remained active for up to 30 days before and one day after the anchor event. Completion within this window was required for the series to continue; patients missing baseline surveys were automatically excluded from subsequent follow-ups. Patient-reported outcome measures were collected using multiple modalities integrated within the EHR, including the patient portal, Interactive Voice Response (IVR) telephone calls, and in-person completion via Welcome Tablets during outpatient visits. These collection methods functioned in parallel, with responses from any source automatically ingesting into the same EHR location for provider review. Longitudinal follow-up responses were predominantly captured via the patient portal.

For longitudinal survey collection, six automated reminders were issued over a 3-week period, at which time the automated response window expired. Manual survey assignment was possible after the 3-week survey period but lacked reminder functionality and responses could not be backdated, leading to difficulty in correlating responses received with the procedure of interest. PROMIS scores were extracted using structured SQL queries from the EHR database.

## Feasibility metrics

*Completion rates* were calculated as the total number of series responses (numerator) divided by the total number of series assignments (denominator). A series assignment is defined as the bundle of all 9 questionnaires assigned at any given timepoint. A successful completion was defined as receipt of responses meeting the study’s minimum required domains—PROMIS Physical Function, PROMIS Pain Interference, and PROMIS Pain Intensity—all of which were necessary for a survey to be considered complete.

*Longitudinal completeness* was defined as the percentage of patients who completed all four timepoints of interest (baseline, 3, 6, and 12 months). This study focused on baseline, 3-month, 6-month, and 12-month; however, the program assigns questionnaires at additional timepoints beyond that as well (2-years, 3-years, 4-years, and 5-years). The automation within the EHR does not require a patient to respond to every timepoint they are assigned. If the patient fails to complete the 3-month assignment, for example, they will still be assigned the 6-month timepoint. Longitudinal completeness measures how well a patient completes multiple intervals over time and aids in assessing the need for additional reminders or engagement with the patient during their longitudinal series.

*Longitudinal engagement* was defined as the percentage of patients who completed all timepoints they were assigned so far (baseline, 3, 6, and/or 12 months). At the time of the data analysis, some patients had not reached all timepoints *yet*. The study timeframe was from 2022 to 2025 and some 2025 series assignments had not yet reached the 6-month or 12-month collection period. Longitudinal engagement measured only the timepoints that were reached and assigned, ensuring that we didn’t inadvertently penalize a patient for not completing a timepoint when they couldn’t have completed it (because they hadn’t received it yet). Longitudinal engagement aids in properly counting (or not counting) responses and avoids inappropriate decision-making if a lack of response was due to not yet being assessed.

*Failure modes* were categorized qualitatively based on process mapping with clinic staff and EHR analysts to identify the causes of non-completion, including EHR limitations, portal inactivity, and lack of staff oversight.

**Fig. 1:**
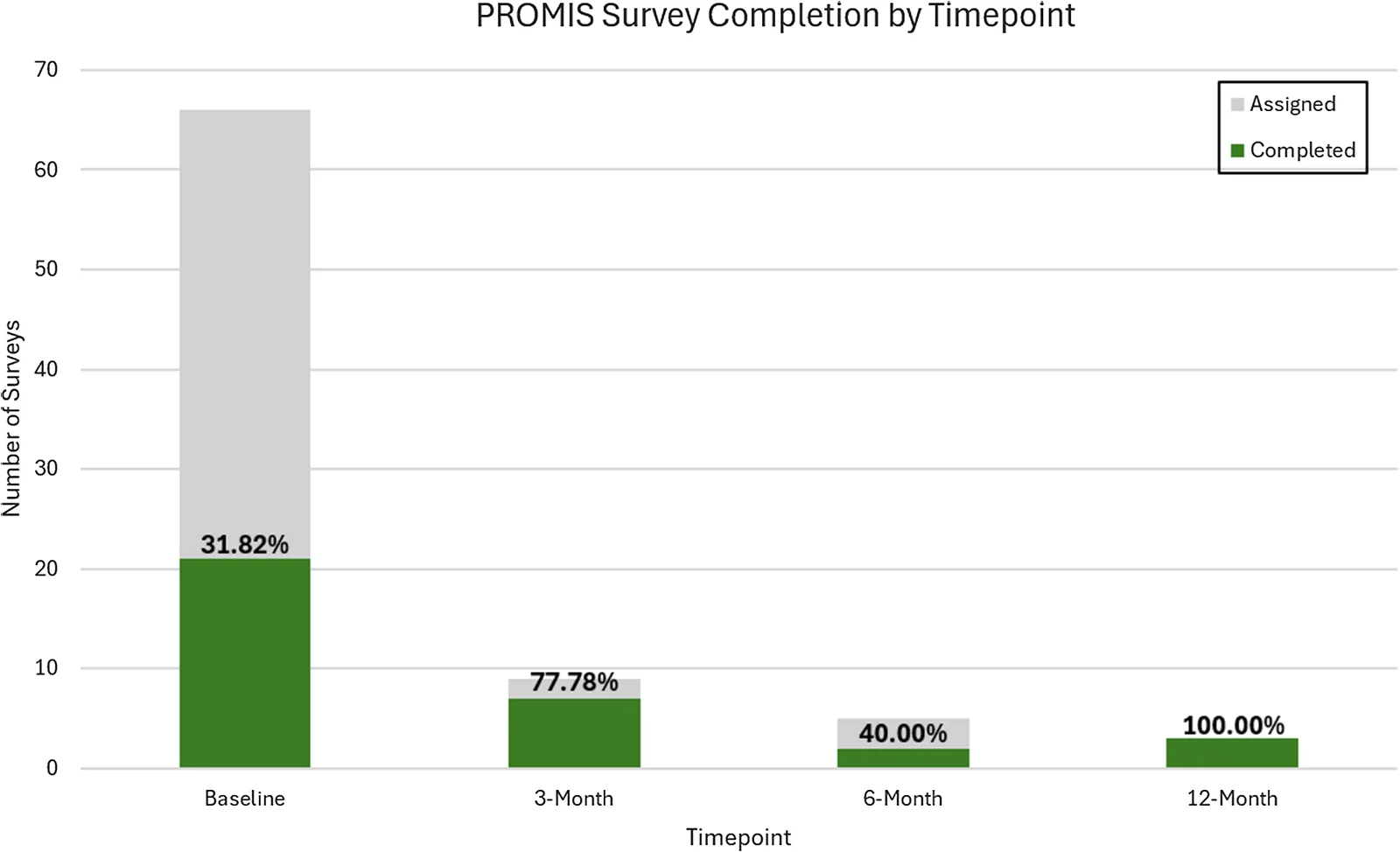
PROMIS completion rates by timepoint. Percentages reflect completed surveys divided by assigned series at each follow-up interval

Completion rates were visualized in Fig. 1 and demonstrated 31.82%, 77.78%, 40% and 100% completion rate at baseline, 3-month, 6-month, and 12-month post survey. Importantly, because completion of each survey was required to trigger subsequent follow-up surveys within the EHR workflow, follow-up completion rates were calculated from a progressively smaller subset of patients, which explains the observed 100% completion rate at 12 months despite substantially lower baseline completion rates.

## Workflow review

Process mapping was conducted with Pain Department clinic staff and EHR analysts to identify points of failure. Data were reviewed from institutional dashboards and cross-checked with EHR logs. Following process mapping, root-cause analysis, and stakeholder feedback, multiple workflow interventions were implemented during the study period.

## Quality improvement intervention

**Fig. 2:**
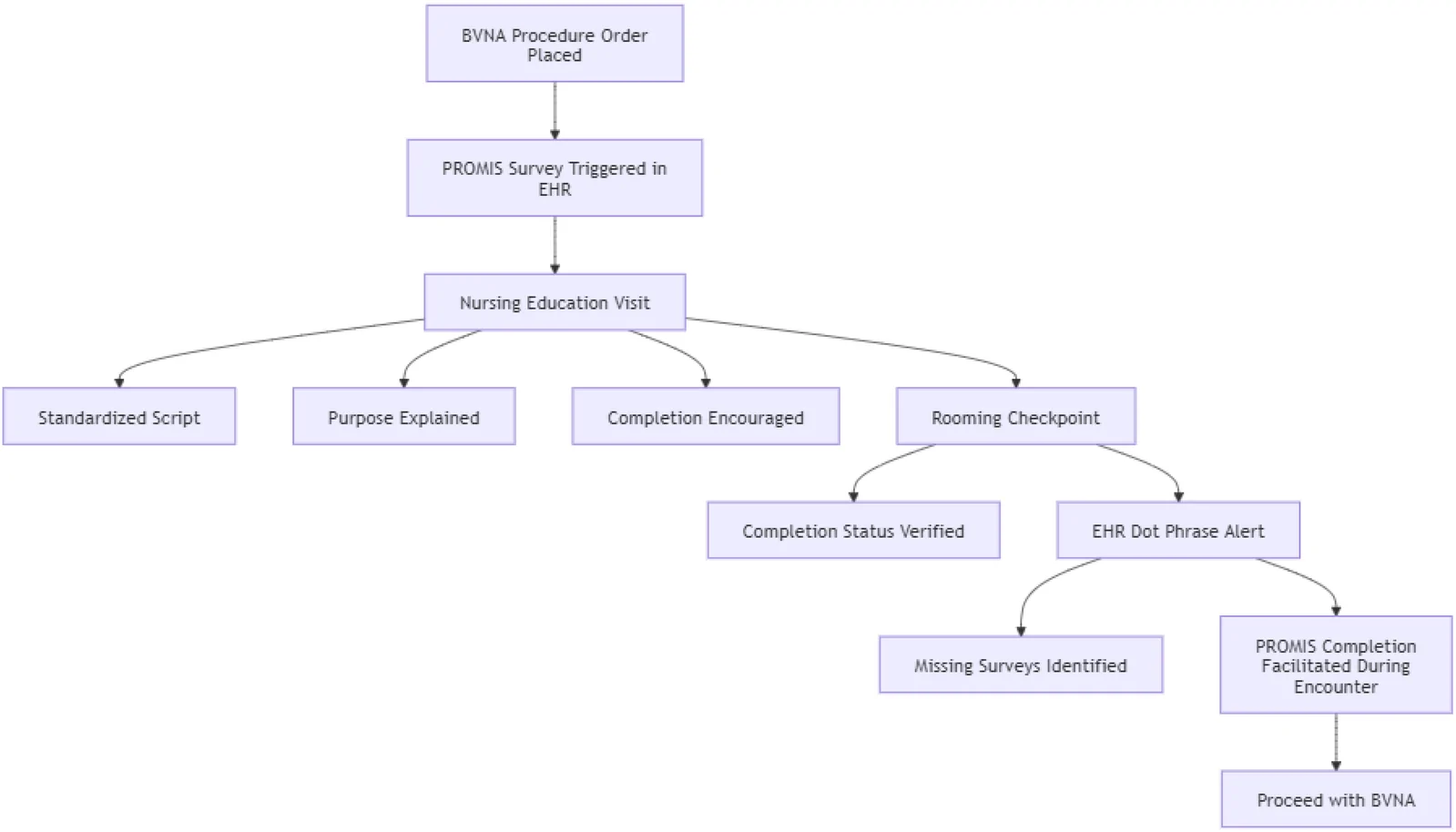
Workflow diagram

**Fig. 3:**
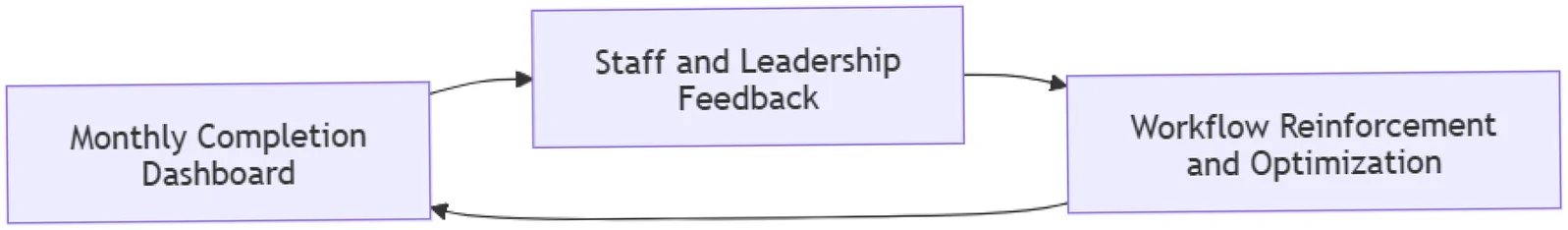
Workflow monitoring

Based on findings from process mapping, root-cause analysis, and stakeholder feedback, a bundled workflow intervention was implemented to address the primary drivers of incomplete PROMIS capture. The intervention targeted three modifiable process gaps: lack of in-person prompting, absence of standardized workflow accountability, and limited visibility into survey completion status (Fig. 2). First, a standardized nursing rooming checkpoint was added to the pre-procedure workflow requiring verification of PROMIS survey completion prior to basivertebral nerve ablation. Nursing staff were provided with brief scripted language explaining the purpose of PROMIS surveys and encouraging patient participation during pre-operative nursing education visit. Second, a real-time EHR visual prompt was implemented through a dot phrase that alerted staff when required PROMIS surveys had not been completed, allowing surveys to be addressed during the patient encounter. Third, monthly site-level completion dashboards were distributed to clinical staff and leadership to provide performance feedback and reinforce accountability (Fig. 3). The workflow changes were initially piloted at a high-volume site and refined through staff feedback before expansion across additional health system locations. The intervention was intentionally implemented as a bundled package to improve workflow reliability and baseline survey capture rather than to isolate the effect of individual components.

## Results

### Baseline PROMIS completion rates

A total of seventy-one unique patients (73 BVNA procedures) were included in the analysis. PROMIS completion rates were 31.82% at baseline, 77.78% at 3 months, 40% at 6 months, and 100% at 12 months among patients who reached those respective timepoints. Longitudinal engagement, defined as patients who completed all follow ups they were assigned so far, was 56%, whereas longitudinal completeness, defined as patients who completed all four timepoints of interest (baseline, 3-, 6-, and 12-month), was 33%. The apparent discrepancy arises because some patients advanced to later timepoints despite missing earlier follow-ups, and thus not all who completed the 12-month survey had complete interim data. Workflow analysis identified several key barriers contributing to gaps in data completeness. The automatic halting of follow-up assignments did not cause missed surveys but reduced the number of patients entering longitudinal follow-up, thereby limiting the denominator for longitudinal analyses. Greater baseline capture would have proportionally increased follow-up participation over time. With this in mind, baseline capture became the point of emphasis for failure mode analysis.

### Failure mode analysis

Primary barriers to completing baseline PROMIS data capture appear to stem from provider awareness, patient engagement, and EHR system limitations. Provider awareness most strongly influenced baseline completion, as the importance of educating the patient on the availability of the PROMIS series was often underrecognized. Patient engagement affected response rates across all timepoints, with varying levels of participation in longitudinal follow-up. Finally, EHR constraints, particularly the inability to assign a Welcome Tablet within a surgical-based EHR module that did not integrate with Welcome, further hindered baseline survey collection. Together, these factors contributed to gaps in data completeness that reflect workflow and system challenges rather than a lack of patient willingness to participate.

### Effect of the workflow intervention on baseline PROMIS completion

**Fig. 4:**
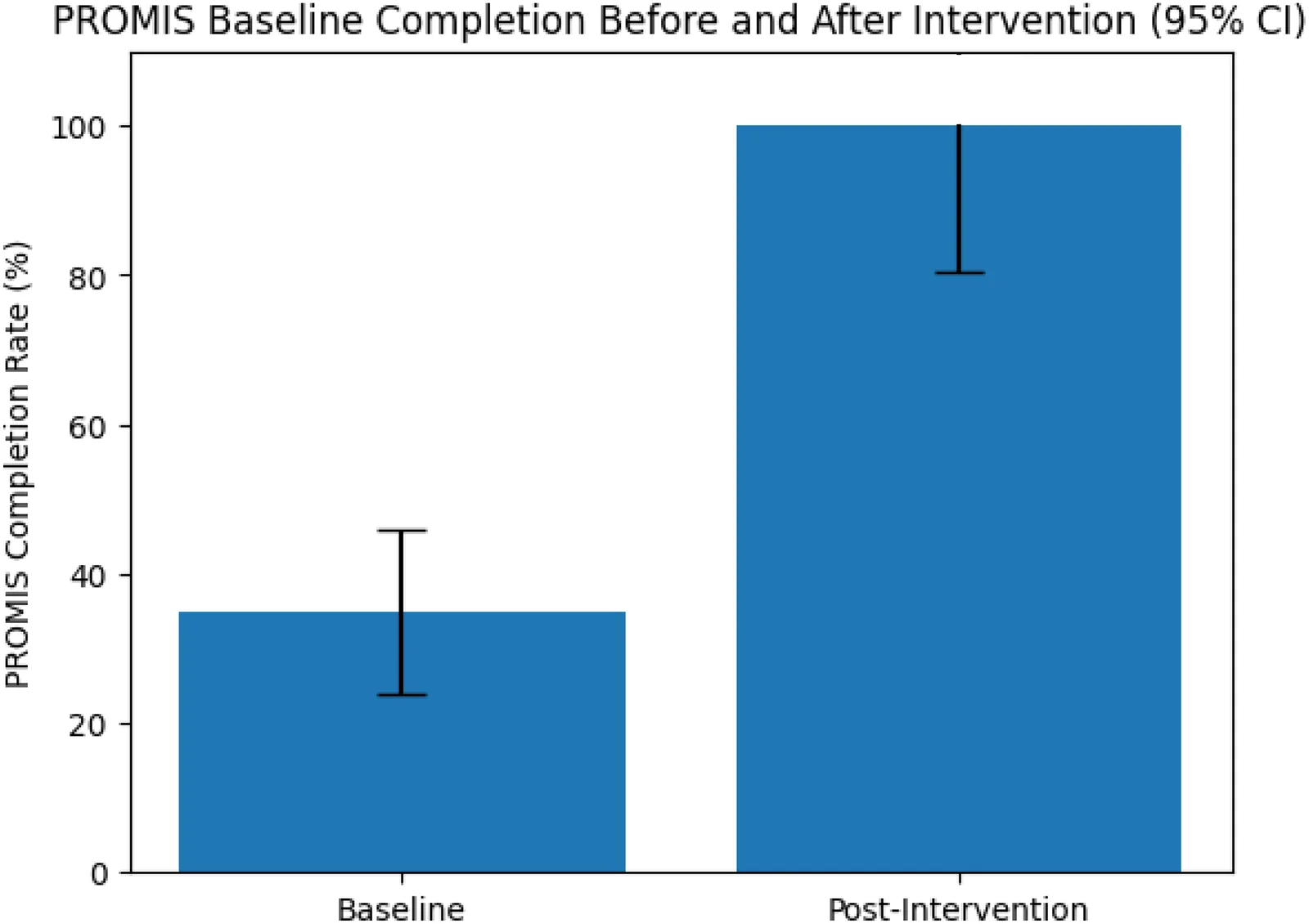
PROMIS baseline completion rate post-intervention

Prior to implementation of the workflow intervention, baseline PROMIS completion among patients undergoing basivertebral nerve ablation was 31.82% (21/66 series assignments). Following implementation of the standardized rooming checkpoint, staff scripting, Epic visual prompts, and monthly performance dashboards, baseline completion increased to 100% during the initial post-intervention observation period (6/6 patients) over three consecutive months (Fig. 4).

Given the small post-intervention sample size and absence of incomplete surveys in the post-intervention cohort, Fisher’s exact test was used to compare baseline PROMIS completion rates before and after implementation of the workflow intervention. Baseline completion improved from 31.82% (21/66) before intervention to 100% (6/6) following implementation, representing a statistically significant improvement in PROMIS capture (*p* = 0.0019).

These interventions and resultant data allow us to explore the central hypothesis identified during root-cause analysis: failure to capture the baseline survey prevents downstream surveys from triggering within the EHR PROMIS surgical series logic. Consequently, improvements in baseline capture directly expand the denominator of patients eligible for longitudinal outcome measurement. Subsequent publications will evaluate the validity of this hypothesis as longitudinal data post-intervention result at 3, 6, 9, and 12 months post-intervention.

### Balancing measure (Clinic workflow efficiency)

To ensure that the intervention did not adversely affect clinical workflow, clinic visit duration during the nurse education encounter was tracked as a balancing measure. Baseline clinic scheduling consisted of 30-minute visit slots.

**Fig. 5:**
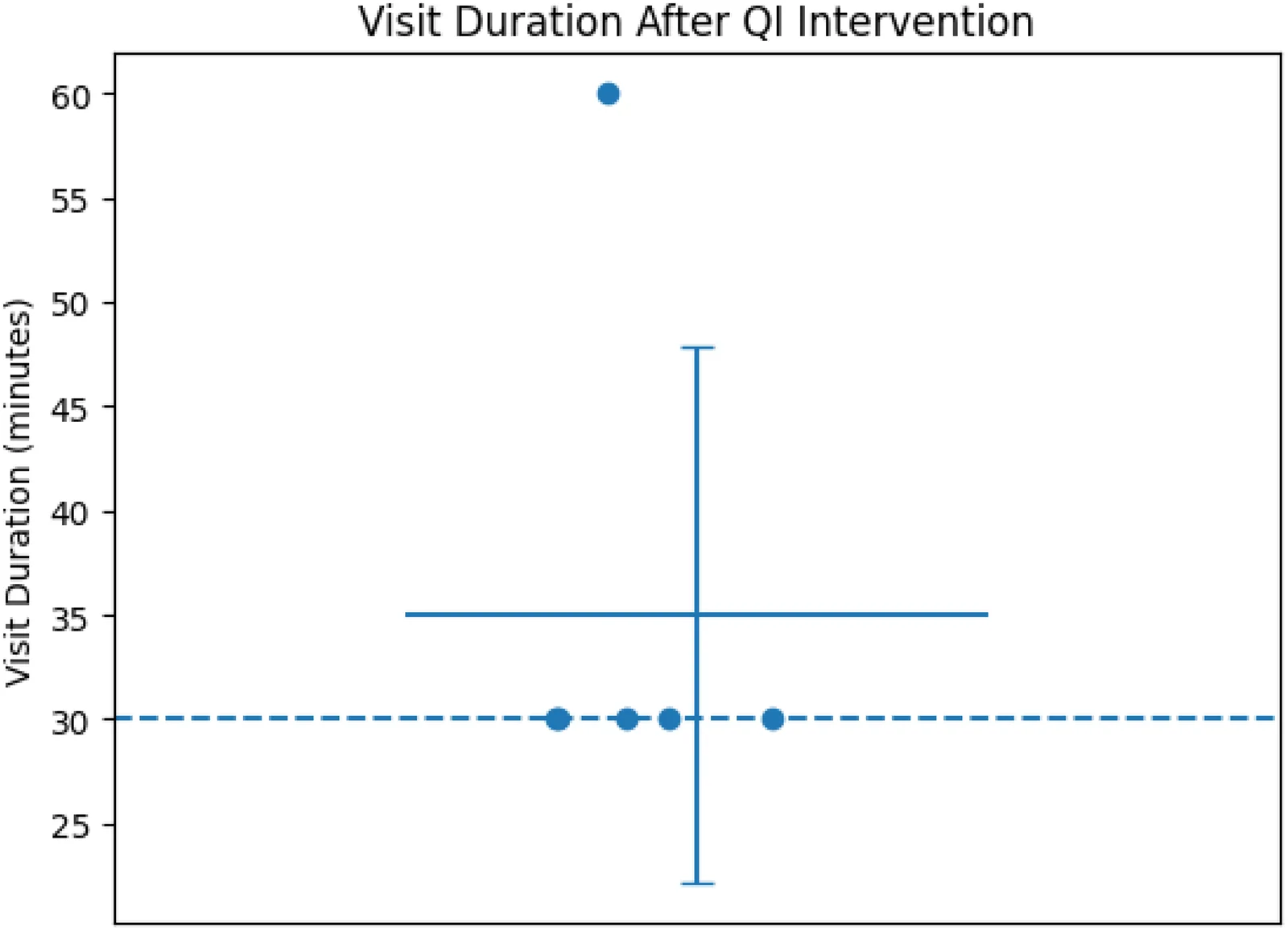
Countermeasure analysis. Horizontal bar represents mean, vertical bar represents 95% confidence interval

Following implementation of the PROMIS workflow intervention, visit duration was measured in six consecutive BVNA encounters (Fig. 5). Five visits lasted 30 min and one visit lasted 60 min, yielding:

- Mean visit duration: 35 min.
- Median visit duration: 30 min.
- Visits remaining within the scheduled 30-minute slot: 83% (5/6).

A one-sample t-test comparing the observed visit duration to the expected 30-minute visit length demonstrated no statistically significant difference (t(5) = 1.0, *p* = 0.36). Given the small sample size, this balancing-measure analysis was exploratory. Five of six visits remained within the scheduled 30-minute slot, and median visit duration was 30 min, suggesting no clear signal of clinically meaningful workflow burden during initial implementation.

## Discussion

This study demonstrates that EHR-embedded PROMIS data collection is feasible within an interventional spine workflow but limited by early attrition and engagement dependencies. Consistent with a quality improvement framework, this study combined baseline feasibility assessment with iterative workflow redesign to improve PROMIS capture. Baseline completion emerged as the primary determinant of longitudinal completeness, as the EHR’s survey logic automatically discontinues follow-up deployment when baseline surveys are incomplete. This design safeguards data quality but also suppresses downstream capture, producing a “front-end attrition effect” driven by workflow gaps rather than patient disengagement. The variability in completion rates reflects the mismatch between automated PROMIS infrastructure and the procedural nature of BVNA, where patients often bypass traditional follow-up encounters that trigger survey delivery. Identified barriers including inactive patient portals and lack of defined staff oversight underscore workflow, patient and provider engagement barriers, rather than system architecture challenges. To improve completeness, institutions should implement hybrid collection models that combine automated EHR deployment with targeted coordinator outreach, assign process ownership for PROMIS monitoring, and integrate completion metrics into provider dashboards to promote accountability. Collectively, these results suggest that optimizing PROMIS integration in interventional pain practice may require re-engineering workflows to prioritize baseline capture, ensuring a multi-method approach in follow-up collection mechanisms, and balancing automation with human oversight.

Importantly, this feasibility analysis occurred alongside active workflow redesign. Identified gaps in baseline survey capture directly informed the implementation of standardized rooming workflows, real-time EHR prompts, and feedback dashboards. These pragmatic interventions specifically targeted front-end attrition while avoiding increased nursing burden, demonstrating that meaningful improvements in PROMIS reliability can be achieved within routine procedural workflows.

A key limitation of this quality improvement initiative is the small post-intervention sample size. While baseline PROMIS completion improved from 31.8% to 100% following implementation of the intervention, only six patients were included in the post-intervention analysis. As a result, the observed statistical significance should be interpreted with caution, as small sample sizes may overestimate intervention effects and limit the reliability and generalizability of findings. These results should therefore be considered preliminary and hypothesis-generating rather than definitive evidence of durable intervention effectiveness. Additional data collection over a longer time horizon will be necessary to confirm the persistence and reproducibility of the observed improvement.

An additional limitation is the generalizability of these findings. This study was conducted within a single health system utilizing a specific Epic-based electronic health record infrastructure and PROMIS deployment workflow. As a result, the identified barriers, workflow dependencies, and effectiveness of the implemented interventions may not be directly applicable to other health systems, procedural practices, or EHR platforms with different survey architectures, staffing models, or patient engagement processes. Future studies across diverse clinical settings and EHR environments are needed to determine the broader applicability and scalability of these findings.

Sustainability of these improvements will depend on continued workflow adherence, staff engagement, and integration of PROMIS monitoring into routine clinical performance feedback.

## Conclusion

EHR-embedded PROMIS data collection during interventional pain procedures is feasible and can be reliably integrated into routine clinical workflows when supported by targeted quality improvement interventions. Implementation of standardized rooming checkpoints, staff education, real-time EHR prompts, and performance feedback mechanisms are potential interventions to improve baseline survey capture and longitudinal data completeness without increasing clinical burden. Baseline survey completion emerged as a modifiable determinant of sustained PROMIS engagement.

These findings demonstrate that hybrid implementation strategies combining automated EHR deployment with structured human oversight can overcome prior workflow and engagement barriers and enable scalable, high-quality patient reported outcome collection in interventional spine care.

## Data Availability

The datasets generated and/or analyzed during the current study are not publicly available due to institutional policies and the inclusion of protected patient health information but may be available from the corresponding author on reasonable request and with appropriate institutional approval.

## **Abbreviations**

BVNA: Basivertebral nerve ablation
BVN: Basivertebral nerve
PROMIS: Patient-Reported Outcomes Measurement Information System
